# Printable biomaterials for 3D brain regenerative scaffolds: An in vivo biocompatibility assessment

**DOI:** 10.1016/j.reth.2025.08.008

**Published:** 2025-08-19

**Authors:** Maylis Combeau, Nina Colitti, Julien Clauzel, Franck Desmoulin, Adrien Brilhault, Juliette Fitremann, Mickaël Chabbert, Matthew L. Becker, Sébastien Blanquer, Lorenne Robert, Melissa Parny, Isabelle Raymond-Letron, Carla Cirillo, Isabelle Loubinoux

**Affiliations:** a Univ Toulouse, Inserm, ToNIC, Toulouse, France; b SOFTMAT Chemistry of Colloids, Polymers & Complex Assemblies, CNRS, Toulouse, France; c Duke Mechanical Engineering & Material Science, Duke University, Durham, NC, USA; d ICGM, University of Montpellier, CNRS, ENSCM, Montpellier, France; e LabHPEC, Université de Toulouse, ENVT, Toulouse, France; f Institut Restore, Université de Toulouse, CNRS U-5070, EFS, ENVT, Inserm U1301, Toulouse, France

**Keywords:** 3D printing, Scaffold, Tissue bioengineering, MRI, Brain repair

## Abstract

**Background:**

Brain regeneration after injury is a challenge being tackled by numerous therapeutic strategies in pre-clinical development. There is growing interest in scaffolds implanted in brain lesions. Developments in 3D printing offer the possibility of designing complex structures of varying compositions adapted to tissue anatomy.

**Methods:**

This feasibility study assessed the cerebral biocompatibility of four bioeliminable Digital Light Processing (DLP) printed materials in the rat model: gelatin methacrylate (GelMA), poly(ethylene glycol)diacrylate (PEGDA) mixed with GelMA (PEGDA-GelMA), poly(trimethylene carbonate) trimethacrylate (PTMC-tMA) and an ABA triblock copolymer of polypropylene fumarate-b-poly γ-methyl ε-caprolactone-b-polypropylene fumarate (P(PF-MCL-PF)). Their tolerance was compared to that of polydioxanone Ethicon (PDSII), a neurosurgery suture component commonly used in clinical practice. A one-month MRI and behavioral follow-up aided in safety assessment.

**Results:**

High-resolution T2 MRI imaging effectively captured the scaffold structures and demonstrated its non-invasive utility in monitoring degradability. PDSII served as a control of the acceptable inflammatory response to implantable foreign bodies. GelMA, PEGDA-GelMA and PTMC-tMA did not affect the permissive glial barrier, promoted cell migration, and neovascularization without additional perilesional microglial inflammation (median mean of 6.5 %, compared to 8.2 % for the PDSII control). However, the GelMA scaffold core was not colonized and allowed a limited neuronal progenitors recruitment. The rigidity of PTMC-tMA facilitated insertion, but posed histological issues. The brain hardly reacted to the P(PF-MCL-PF).

**Conclusion:**

All these materials can serve as a basis for brain regeneration. PEGDA-GelMA emerged as a promising candidate for intracerebral implantation, combining biophysical and bioprinting advantages while maintaining an acceptable level of inflammation compared with clinically used suture, paving the way for innovative therapies.

## Highlights

• The best candidate must be chosen in comparative studies on different biomaterials.
• An acceptable level of inflammation is necessary to rank degradable biomaterials.
• Neurosurgery sutures commonly used in clinical practice can serve as gold standards.

## Graphical abstract

**Figure:**
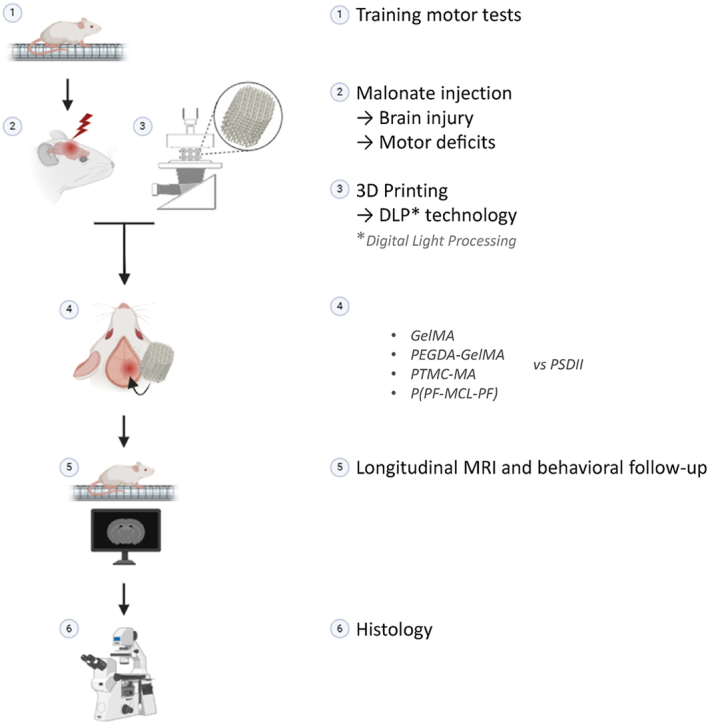

## Abbreviations

ARRIVE: animal research: reporting of *in vivo* experiments
BAPO: phenylbis(2,4,6-trimethylbenzoyl)phosphine oxide
BSA: bovine serum albumin
CAD: computer-aided design
DAB: 3,3′-diaminobenzidine
DAPI: 4′,6-diamidino-2-phenylindole
DCX: double cortine
DLP: digital light processing
EDTA: ethylenediaminetetraacetic acid
ENVT: ecole nationale veterinaire de toulouse
Gal-C9: *N*-nonyl-d-galactonamide
GelMA: gelatin methacrylate
GFAP: glial fibrillary acidic protein
HE: hematoxylin-eosin
HMB: 2-hydroxy-4-methoxybenzophenone
IMFT: institut mécanique des fluides de toulouse
MRI: magnetic resonance imaging
NSS: neurological severity scale
P(PF-MCL-PF): poly(propylene fumarate-b-γ-methyl-ε-caprolactone-b-propylene fumarate)
PBS: phosphate-buffered saline
PCL: poly(caprolactone)
PDSII: polydioxanone ethicon
PFA: paraformaldehyde
PEGDA: poly(ethylene glycol) diacrylate
PTMC-tMA: methacrylate end-functionalized-poly(trimethylene carbonate)
ROI: region of interest
SVZ: subventricular neurogenic niches
TPO: diphenyl(2,4,6-trimethylbenzoyl)phosphine oxide
UV: ultra-violet
3D: three dimensions

## 1 Introduction

Brain regeneration is a very challenging research domain. Following an acute injury like stroke or traumatic brain injury, the brain requires structural and functional tissue regeneration, with the integration and survival of new neurons into existing circuits. Although cerebral plasticity can initiate regeneration processes to a certain extent, these are not sufficient to make up for massive losses of neurons. In recent years, significant advances in biomaterials research have made tissue engineering a promising approach to induce brain regeneration [[1], [2], [3], [4], [5]].

Computer-aided design (CAD) [6] enables the development of complex three-dimensional (3D) scaffolds, which can be modelled after tissue anatomy. In the field of brain repair, advanced biomimetic materials play a crucial role by providing structurally optimized environments that support neural regeneration. Specifically, these materials can be engineered with defined architectures that facilitate and direct axonal growth *in situ* [7], offering a distinct advantage over conventional unstructured biomaterials.

For an effective therapeutic strategy, the scaffold must be a reliable, non-toxic device, biocompatible with the host tissue. Furthermore, to optimize recovery and ensure complete recolonization of the damaged area, it is crucial to use a long-term resorbable material, which enhances the efficacy of biomedical scaffolds.

In a previous methodological study, we explored the strategy of combining 3D printing by Digital Light Processing (DLP) with implantable biomaterials for brain tissue repair [8]. In particular, we investigated the response to four different biomaterials using a rat model of acute brain injury. Among several biomaterials including poly(ethylene glycol) diacrylate (PEGDA), poly(caprolactone) (PCL), and PEGDA mixed with gelatin methacrylate (GelMA), the latter, showed promising as a potential material for supporting brain regeneration [8].

Since PEGDA showed poor biocompatibility, we decided to assess GelMA alone in the present *in vivo* study. However, GelMA suffer from recognized low mechanical properties, which will be a serious limitation in the procedure of brain tissue engineering and thus it will be compared with a copolymer formed from PEGDA and GelMA. In this investigation, we also compare the performance of DLP printed scaffold based on GelMA biomaterial with three distinct photo-crosslinked synthetic biomaterials recognized for their outstanding mechanical properties, namely methacrylate end-functionalized-poly(trimethylene carbonate) (PTMC-tMA) and poly(propylene fumarate-b-γ-methyl-ε-caprolactone-b-propylene fumarate) (P(PF-MCL-PF)).

Gelatin derived from natural animal sources, has been shown to promote cell adhesion thanks to its RGD sequences [9], which makes it an interesting candidate for tissue regeneration. Incorporating PEGDA into a gelatin matrix, on the other hand, is particularly well suited to enhancing photo-polymerization-based printing, not only by improving printability, but also by contributing to structural and mechanical stability by adjusting the PEG type, while maintaining bioactivity and elimination [[10], [11], [12]].

PTMC-tMA and P(PF-MCL-PF) are synthetic biomaterials that have been already used in 3D printing based photopolymerisation process and already investigated in tissue engineering of soft tissue [13,14]. PTMC material has been greatly tested in biomedical device *in vivo*, for instance as suture threads for neurosurgery [15], subcutaneous device [16], or eye implant [17]. Once end-functionalized with photo-polymerisable moieties such as methacrylate, PTMC-methacrylated is an emerging photoprintable biomaterial, already used as designed scaffold in orthopedic reconstruction [18], or in the context of peripheral nerve repair to guide nerve regeneration *in vitro* [19]. To the best of our knowledge, no *in vivo* study has yet investigated the use of photoprinted PTMC-tMA for brain regeneration. On its side, the P(PF-MCL-PF) is a non-cytotoxic polymer according to ISO 10993-5, and is well tolerated *in vivo* [20,21]. It undergoes biodegradation through hydrolysis of its ester bonds [22,23], producing propylene glycol and fumaric acid [18, 19]. Notably, it also offers the great advantage of being printable without requiring pre-methacrylation [[24], [25]]. To date, no *in vivo* studies have investigated the use of photochemically printed P(PF-MCL-PF) for brain regeneration. In addition to the different biomaterials aforementioned, and as previously done [16], we used Polydioxanone Ethicon (PDSII), a degradable suture thread commonly used in neurosurgery. This served as a reference to evaluate the expected biocompatible response following brain injury and the implantation of foreign material in both healthy and lesioned sites. PDSII was intended for incorporation into a *N*-nonyl-d-galactonamide hydrogel [26].

GelMA, PEGDA-GelMA, PTMC-tMA, P(PF-MCL-PF) have been used as a printable resin to fabricate by Digital Light Processing (DLP) designed scaffolds suitable for brain repair. These scaffolds were implanted in a rat model of brain injury following a model procedure, validated by our group [27]. Typically, the procedure consists in the injection of a toxin, malonate, into the motor cortex, resulting in extensive tissue damage and consequent functional deficits. The primary goal of this study was to evaluate the feasibility and safety of various candidate scaffolds. Secondary objectives included the characterization of cell types present around the scaffold in the weeks following implantation and analyzing the nature of the tissue responses occurring around and within the scaffold.

## 2 Material and methods

### 2.1 Scaffolds design

The scaffolds were designed using Autodesk Fusion 360® computer-aided design software. Structurally, the aim was to maximize porosity, cellular infiltration and 3 orthogonal preferred directions. The design had to be as ergonomic as possible, accommodating intracerebral insertion through a maximum 5 mm diameter cranial flap, and adjusted to lesion volumes with inter-individual variability. Lesion volumes were calculated from post-injury T2 MRI scans (details below).

An optimized CAO model designed by our team was presented in a recent study [8]. This model consists of two different parts (Fig. 2): i/a handle at the top, for manipulating the scaffold, ii/then porous scaffold displaying channels and pillars with different sizes (Table 1). The handle is cut at the time of insertion and is not intended to remain in the brain.

**Table 1:** Scaffolds characteristics.

Biomaterials	Mesures	Channels (μm)	Pillars (μm)
PEGDA-GelMA	Theoretical	300	400
	Real	335 ± 35	473 ± 58
GelMA	Theoretical	300	400
	Real	225–310	450–530
PTMC-tMA	Theoretical	300	400
	Real	283 ± 17	224 ± 13

Outer volume of the scaffolds is given as well as dimensions (Width∗Length∗Height). There is personalisation to the lesion volume for the PEGDA-GelMA.

This refined design was intended to be the printing template for the different biomaterials presented below. However, due to the intrinsic properties of the printing resins, it was not possible to achieve the planned spatial resolution with all of them. For each biomaterial, theorical dimensions, as defined in the model, are reported in Table 1 along with the real dimensions, measured after printing. GelMA and PEGDA-GelMA had a high viscosity and were printed with lower pattern resolution than PTMC-MA (Fig. 2, Table 1). As for P(PF-MCL-PF), the ethyl acetate solvent used to formulate the resin evaporated rapidly, which meant that we had to print thin sheets of copolymer in a relatively short time. A porous scaffold could therefore not be achieved with this material (Suppl. Fig. 1).

### 2.2 Scaffold preparation

The scaffolds were printed using a LumenX™ (CellInk Inc., Göteborg, Sweden). This 3D printer relies on DLP (digital light processing) technology, with a wavelength of 405 nm (violet) and a horizontal resolution of 50 μm (x, y).

⁃ Gelatin Methacrylate (GelMA)

GelMA Photoink ™ (CellInk Inc.) is a ready-to-use solution (Table 3). GelMA stands for Gelatine Methacrylate, a type A gelatin of strength 300 g Bloom that can be photo-crosslinked. We used the printing parameters recommended by the manufacturer: a layer height of 50 μm and a light power of 20 mW/cm^2^. Exposure times for the two first layers were set at 14 s to ensure adhesion to the plate, and subsequent layers had exposure times of 7 s. GelMA modulus is around 22 ± 5 kPa (CellInk).

⁃ Polyethylene Glycol Diacrylate (PEGDA) mixed with GelMA (PEGDA-GelMA).

**Table 2:** Volume and intensity of scaffold.

T2 MRI values of scaffolds		Intensity		Volume (mm3)		Volume loss after implantation (%)
		Post -implantation	3 weeks post - implantation	Post -implantation	3 weeks post - implantation	
GelMA	**Mean** ± **SD**	**2,33** ± 0,19	**2,00** ± 0,14	**31,29** ± 0,52	**30,34** ± 1,15	**48** ± 1
PEGDA-GelMA		**2,22** ± 0,25	**1,93** ± 0,01	**59,99** ± 8,07	**66,22** ± 11	**19** ± 21
PTMC-tMA		**0,58** ± 0,19	**0,68** ± 0,19	**55,90** ± 2,69	**59,85** ± 8,27	**14** ± 4

Data were measured on T2-weighted images post-implantation and 3 weeks after for the GelMA (n = 2), PEGDA-GelMA (n = 2) and PTMC-tMA scaffolds (n = 2). Intensities are normalized by the mean intensity in the contralateral healthy cortex. The volume loss after implantation was calculated by the ratio between the T2 volume post-implant and the real volumes post-printing after swelling.

**Table 3:**
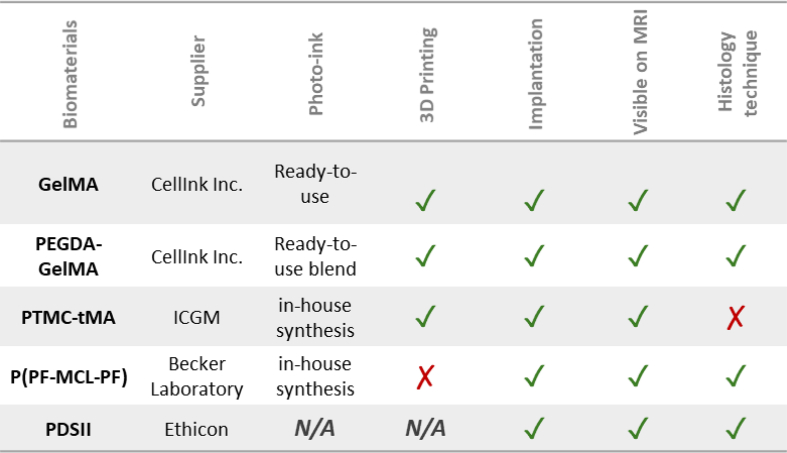
Summary table of biomaterial validation at different critical stages of the protocol.

Legend: **✓** validated step; ***✗*** step not validated; *N/A not applicable*.

GelMA and PEGDA-200 Photo Ink™ (CellInk Inc.) were combined in equal proportions. Both inks were warmed separately in water at 37 °C before mixing. The photoinks are provided as ready-to-use solution, without additional compounds for printing. Printing with LumenX was carried out with a layer thickness of 50 μm, an exposure time for the first layers of 15 s, and 5 s for subsequent ones, a light output of 20 mW/cm^2^, and the printer's internal heating set to 75 °C. PEGDA 200 modulus is around 200 ± 20 kPa (CellInk).

⁃ Poly(Trimethylene Carbonate)-triMethacrylate (PTMC-tMA)

PTMC-tMA was previously synthesized according to published protocols [16], and was provided in a paste-form. For printing, the PTMC-tMA resin was first dissolved in propylene carbonate (TCI, France; 50 % w/w of the total solution), a biosourced solvent with low toxicity and high boiling temperature to avoid evaporation during DLP printing. Photoiniator TPO (Diphenyl(2,4,6-trimethylbenzoyl)phosphine oxide, Sigma Aldrich, Saint-Quentin-Fallavier, France; 4 % w/w) and photoabsorber Orange G (Sigma Aldrich; 0.3 % w/w) were then added in the mixture to final formulation. Printing with LumenX was carried out with an exposure time of 3.75 s on the first layers, 1.25 s on subsequent layers, a light output of 10 mW/cm^2^ and the printer's internal heating set to 50 °C. After printing, the scaffolds were rinsed in propylene carbonate (4 washes, changing twice a day). The low-volatility propylene carbonate was extracted with 6 additional washes of acetone over 3 days, after which the acetone was dried. PTMC-tMA modulus is around 1–10 MPa [28,29].

⁃ Poly(propylene fumarate-b-γ-methyl-ε-caprolactone-b-propylene fumarate) (P(PF-MCL-PF))

The P(PF-b-γMeCL-b-PF) copolymer [5:30:5] was obtained in the form of a viscous liquid, and was mixed with ethyl acetate in a copolymer:solvent weight ratio of 60:40 wt%, following a previously reported protocol [30]. The crosslinking agent was trimethylolpropane tris(3-mercaptopropionate) (thiol), in a 10:1 alkene:thiol ratio. The photoinitiator was phenylbis(2,4,6-trimethylbenzoyl)phosphine oxide (BAPO), 0.5 wt% by weight based on the copolymer, and the scavenger radical was 2-hydroxy-4-methoxybenzophenone (HMB) 0.3 wt%. To prevent the resin from hardening before printing, thiol was added to the mixture right before printing. Printing with LumenX was carried out with an exposure time of 30 s on the first layers, 10 s on subsequent ones, at a power of 25 mW/cm^2^. Post-curing was carried out by exposing the pieces to UV (λ = 405 nm) with Anycubic Wash and Cure Plus for 30 min. The pieces were rinsed with ethyl acetate and isopropanol, then left to dry for 48 h in an oven at 60 °C.

Due to the speed of evaporation of solvent, the printing resin turned into a highly viscous liquid very fast, making it impossible to control the printing process and to achieve successful and repeatable results. Despite several attempts at changing the printing conditions, we were not able to produce a complete 3D implant matching the theoretical design. We therefore decided to use instead a printed fine slice that was parted in fine slices, as well as a rectangle which was rolled into a 9 mm cylinder, which were then implanted into the lesion (Suppl. Fig. 1). The study around the P(PF-b-γMeCL-b-PF), being based on pieces of non-porous biomaterials, was not comparable with other biomaterial on the criterion of colonization. Data related to the P(PF-b-γMeCL-b-PF) were therefore be presented as supplementary data.

⁃ Polydioxanone Ethicon (PDSII) + *N*-nonyl-d-galactonamide hydrogel

The Gal-C9 hydrogel was manufactured at the SoftMat laboratory [31]. It is an *N*-nonyl-d-galactonamide hydrogel (0.5 wt%, i.e. initially, when not compacted, 2.5 mg of this molecule in 500 μL of phosphate-buffered saline -PBS-). It is an oxidized galactose (galactonic acid) to which the amino fatty chain is grafted, forming an amide with the acid. *N*-nonyl-d-galactonamide was prepared according to the synthesis described in Ref. [32]. This hydrogel was intended to serve as a primary support for the insertion and stabilization of PDSII threads in the lesion.

### 2.3 Scaffold decontamination

Printed scaffolds were subjected to a series of decontamination steps before their implantation *in vivo*. They were rinsed 3 × 10 min with sterile Dulbecco's phosphate-buffered saline and antibiotics-antimycotics (Penicilline-Streptomycine 1 %, PenStrep, Gibco, Thermo-Fisher, Illkirch-Graffenstaden, France). The day prior to implantation, scaffolds were soaked in sterile 70 % ethanol for 2 h at room temperature (RT). Next, scaffolds underwent 3x10-min washes with sterile PBS + PenStrep (1 %, Gibco), and were stored overnight at 4 °C. On the day of implantation, scaffolds were exposed to UV light for 30 min and kept in a sealed environment with the same PBS + PenStrep solution. All decontamination procedures, which included ethanol treatment, PBS rinses, and UV exposure, were carried out following the Biosafety Level 2 guidelines.

### 2.4 Animals

The animal cohort is composed of nine female Sprague-Dawley rats, aged 11 weeks and weighing between 280 and 320 g (Janvier Labs, Le Genest-Saint-Isle, France). The sex of the animals had no impact on our previous studies [33]. No sex differences were found in our study model. They were pair-housed in enriched cages measuring 30 cm in length, 18 cm in height, and 32 cm in width, within a controlled environment set at 20 °C and a 12-h light/12-h dark cycle, with free access to food and water. Material enrichment was changed twice a week, and social enrichment was carried out weekly. Social enrichment is achievable with female animals that show no aggressive behavior. All experimental procedures adhered to the guidelines established by the Council of the European Communities (EU Directive 2010/63). The study protocol was approved by the "Direction Départementale de la Protection des Populations de la Haute – Garonne" and the "Comité d’éthique pour l'expérimentation animale Midi-Pyrénées" (protocol n° APAFIS#22419-2019101115259327v5). In accordance with the principles of the 3R (Replacement, Reduction, Refinement), we employed in this pilot study the smallest number of animals necessary. Furthermore, all experiments were conducted in strict compliance with the ARRIVE (Animal Research: Reporting of In Vivo Experiments) guidelines.

All the rats of the study underwent a malonate-induced brain injury. The distribution of rats among experimental groups was as follows: GelMA: n = 2; PEGDA-GelMA: n = 2; PTMC-tMA: n = 2; (P(PF-MCL-PF)): n = 1; PDSII: n = 1; Injured without implant: n = 1. The rats were matched according to the size of the lesions and the group sizes were chosen to facilitate in-depth case studies.

### 2.5 Brain injury

Rats were anesthetized with isoflurane (3 % during induction and 2–3 % for maintenance, delivered at a flow rate of 0.7 L per minute of oxygen). They were then secured on a stereotaxic frame (Bioseb lab, Vitrolles, France). Pre-medication involved an intraperitoneal injection of methylprednisolone (20 mg/kg, Centravet, Castelnaudary, France), and, prior to the scalp incision, a subcutaneous injection of lidocaine 2 % (4 mg/kg, Centravet) was administered for local analgesia.

Cortical lesions were induced in the motor area (M1) by injecting malonate (5 μL, 3 M solution, pH 7.4 in phosphate-buffered saline (PBS); Sigma-Aldrich) at stereotaxic coordinates of 2.5 mm lateral and 0.5 mm anterior to Bregma, and a depth of 2 mm [34]. The hemisphere injured corresponded to the dominant paw of each rat, identified by the grip strength test. This model was previously validated by our team [3,35].

### 2.6 Motor function assessment

Throughout the course of the study, rats’ behavior was monitored to track the emergence of clinical symptoms and potential effects or reactions after the biomaterial implantation. Motor tests were also conducted at different time-points, which included the Grip Strength Evaluation and the Neurological Severity Scale (NSS) [8,35]. The grip strength test assesses the maximal force exerted by the forelimb muscles. Each paw is measured separately. Sensorimotor function was assessed through the NSS, which encompasses five tests that focus on reflexes, stability, sensitivity, and mood. The NSS was scored on a scale ranging from zero to 16 points, with high scores corresponding to more severe deficits.

These tests had been validated by our team in previous protocols [19, 21, 22], and, in order to compare the behavioral metrics obtained in the present work, we used as reference data the scores of eight injured and eight non-injured rats from past studies using identical injury model and kinetics [8,35].

Rat elevation in an open field was assessed during 5 min prelesion and at the end of the experiment.

### 2.7 Implantation

Animals (n = 8) underwent a second surgical session 8 days after injury, a time chosen to align with the peak of neurogenesis [36]. The procedure conditions matched the initial surgery. To insert the scaffolds into the lesioned area, a 5 mm diameter cranial flap was drilled, removed temporarily, and finally placed back at the end of the surgery. For the P(PF-MCL-PF) rat, we tried to fill the lesion with the chips of P(PF-MCL-PF) by first inserting the larger cylindrical piece in the striatum and then filling the space left using plane pieces. Considering that such a difference in the architecture of the P(PF-MCL-PF) scaffold may bias the interpretation of the biocompatibility results, the P(PF-MCL-PF) will not be presented in the following sections, but rather as part of the supplementary materials (Supp. Fig. 1).

**Fig. 1:**
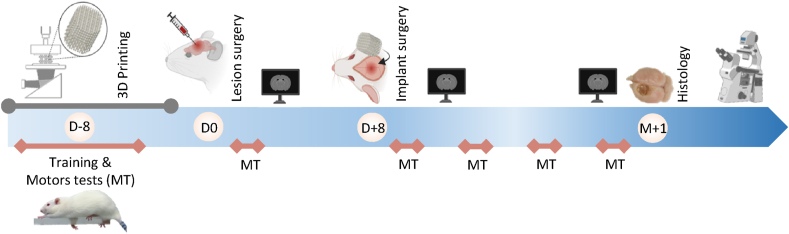
**Study protocol overview.** The time-points shown on the top line illustrate the key experimental steps. 3D printing of biomaterials (grey line): The protocol begins with the optimization of printing methods, followed by the printing and decontamination of scaffolds for *in vivo* implantation. Day zero of the protocol is launched with the injection of malonate for lesion induction. One week later, scaffolds are implanted in the lesion. The bottom line shows the kinetics of the behavioral tests (pink line): for training, tests started before the brain injury and were performed at different times after brain lesion: three days and one, two, three and four weeks. MRI acquisitions (logo screen): three successive MRI sessions: post-lesion = pre-implantation, post-implantation and pre-sacrifice at 1 month. Animal sacrifice: for histological analysis.

For the control rat receiving PDSII + Gal-C9, the Gal-C9 hydrogel crushed and immediately degraded, was not visible on 24h-MRI, nor on 1 month-histology. We will therefore only discuss on PDSII as an inflammation-controlling biomaterial since it was still present on histology, although we recognize that Gal-C9 residues were introduced in the lesion. For the PDSII suture wires, 55 guiding threads of polydioxanone Ethicon® 6-0, between 4 and 6 mm long, have been implanted in the lesion site, with a ventro-dorsal orientation. A comprehensive protocol of the study is described in Fig. 1.

**Fig. 2:**
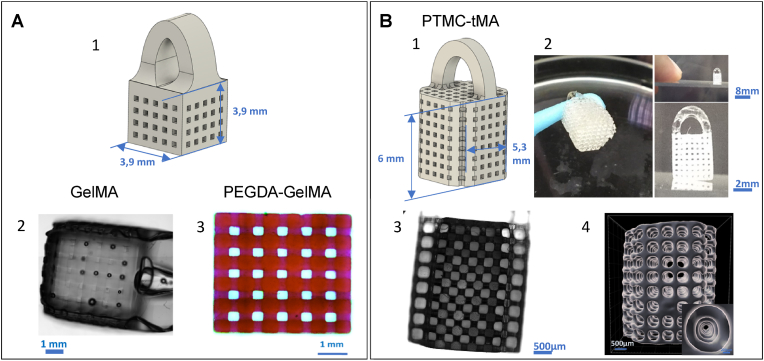
**Scaffold CAD model and design after printing**. A [1]: 3D model for GelMA and PEGDA-GelMA, including a handle on top for handling [2]. image of the structure and pores under a white light microscope x20 [3]. image of Eosin-stained PEGDA-GelMA under a white light microscope x20. B [1]: 3D model for PTMC-MA, including a handle on top for handling. Corners are cut so that the implant can pass through the 5 mm diameter round hole drilled in the skull. The scaffold height without the handle is 6 mm [2]. Printed scaffold held by its handle with custom-made tool (left); Scaffold on a glass slice (top); Close-up on the scaffold (bottom), with visible canals [3]. Side view of the scaffold under a light microscope, with the handle positioned at the top (out of frame). The channels running through the structure are clearly observable, and the bright areas at some of the intersections correspond to points of contact between the scaffold and the glass slide, hence altering the light path [4]. Micro-CT imaging. The size of the scaffold can be adapted to the size of the lesion (model A or B).

### 2.8 In vivo MRI

Imaging was carried out using a 7T preclinical MRI scanner (Biospec 70/16, Bruker, Ettlingen, Germany) equipped with a volume transmit coil and a 2x2 element surface receiver coil. Within the magnet, animals were positioned in a temperature-regulated imaging enclosure to maintain a body temperature at 37 °C. Anesthesia was maintained by administering an Isoflurane/O2 mixture, with the concentration adjusted between 1.5 % and 2.5 % based on the animal's respiratory rate. The following scans were performed: anatomical imaging in T2-weighting using a T2 Turbo-RARE sequence with TE/TR values of 35.75452 ms, producing coronal slices with a spatial resolution of 0.137x0.137 × 0.500 mm^3^; perfusion imaging: cerebral blood flow maps were acquired using pseudo-continuous arterial spin labelling combined with an inversion efficiency measure, in accordance with [37]. Scaffold volume and intensity were recorded by making ROIs with MRIcron. Intensity was normalized by that of ROI in the contralateral healthy cortex [37].

### 2.9 Histology and immunofluorescence

One month after inducing the lesion, the animals received a lethal injection of 1 ml of sodium pentobarbital (159 mg/kg, Centravet) intraperitoneally. To drain blood from the brain vessels, an intracardiac perfusion of heparinized 0.9 % NaCl (200 ml, 20 min) was conducted, followed by a 4 % paraformaldehyde (PFA) perfusion (250–300 ml, 40 min) to fix the tissues. The brain was then extracted and immersed in a 10 % buffered formalin solution for 24 h of post-fixation at 4 °C. It was subsequently embedded in paraffin (at ENVT, France). The paraffin blocks were sectioned using a microtome (Leica SM 2010R, Nanterre, France). Approximately 100 coronal sections, each 8 μm thick, were obtained from each brain. Two out of every twelve sections underwent staining with Hematoxylin Eosin (HE) and Cresyl Violet acetate, following standard procedures. The HE-stained slides were examined under a white light microscope and scanned with a digital slide scanner (Panoramic desk 3D HISTEC). Brain sections corresponding to the lesion site were subjected to immunohistochemical staining with glial fibrillary acidic protein (GFAP 1:50, clone 6F2, ref: MA1-35377 Invitrogen), an astrocytic marker, and Masson's Trichrome (a collagen fiber marker).

For immunofluorescence staining, the section levels were identified using the Paxinos and Watson atlas [34]. Paraffin-embedded sections were unmasked in Tris EDTA buffer (pH9) in the microwave at 300W for 10 min. The labelled slides were placed in a humid chamber at room temperature and incubated for 30 min with a blocking buffer consisting of 1 % bovine serum albumin (BSA, Sigma-Aldrich) and 10 % serum (donkey or goat, Thermo Fisher Scientific or Sigma-Aldrich, respectively) to block non-specific binding sites. The sections were then exposed to primary antibodies for 90 min (Table 4). Following three 10-min washes at room temperature with a washing buffer (PBS 1X + 0.5 % BSA), the sections were incubated with specific secondary antibodies conjugated to a fluorochrome for 50 min, at room temperature and in the dark. After three additional 10-min washes at room temperature with the washing buffer, a fluorescent mounting medium (Fluoroshield™ with DAPI, Sigma-Aldrich) was applied to mount the sections and simultaneously counterstain the nuclei with incorporated 4′,6-diamidino-2-phenylindole (DAPI). Images were captured using a Nikon Eclipse Ti2 series fluorescence microscope and analyzed using Fiji and Imaris image analysis software.

**Table 4:** Immunofluorescence primary antibody list.

Antibody I	Host species	Dillution	References	Labelling
Anti-GFAP	Rabbit	1/500	DAKO:Argilent#Z0334 clone 6F12	mature astrocytes
Anti-Iba1	Rabbit	1/500	WAKO #019-19741	macrophages, monocytes, microglia
Anti-DCX	Rabbit	1/250	Abcam #ab207175	immature and migrating neuroblasts
Anti-DCX	Rabbit	1/500	Wako #019-19741	
Anti-β-3-Tubulin	Rabbit	1/500	Covance#PRB435P	Immature neuron
Anti-NeuN	Rabbit	1:3000	Abcam#ab177487	mature neuron
Anti- Lectin	from Lycopersicon esculentum (tomato) monoclonal conjugate with FITC	1/200	Sigma-Aldrich #L0401	endothelial cells

Cell spreading in the scaffolds was estimated using a grid superimposed on the scaffold sections. An approximate ratio between the number of cells in the grid comprising colonizing tissue and the number of empty cells in the grid was reported. This ratio was calculated over 3 levels of sections for each implant.

### 2.10 Statistical analysis

GraphPad Prism 10 software was used for analyses and graphs design. Data are presented as median and interquartile range. The points shown on the graphs represent microscopic fields on brain slices. Several points on the same axis represent several microscopic fields on the section(s) of a single rat. When the data of two rats of the same group are presented, they are displayed separately on the x-axis, with each field/point corresponding to an individual rat.

## 3 Results

### 3.1 Scaffold characterization

Using an optical microscope, we measured the sizes of the scaffolds after printing (Fig. 2, Table 1).

⁃ Gelatin Methacrylate (GelMA)

The swelling measured in 1D in PBS was 10 ± 3 % (Table 1). The porosity calculated by simulation was 26 %.

⁃ Polyethylene Glycol Diacrylate (PEGDA) mixed with Gelatin Methacrylate (GelMA).

The swelling measured in PBS at room temperature was 17 ± 3 % (Table 1). From these data, porosity was estimated at 40 ± 6 %.

⁃ Poly(Trimethylene Carbonate)-tri-Methacrylate (PTMC-tMA)

The expected shrinkage of the PTMC-tMA scaffold due to the elimination of propylene carbonate solvent, was measured in 1D at 27 ± 1 %, equivalent to a volume loss of 61 % (Table 1). As PTMC-tMA is hydrophobic, size variations related to the presence of water or its osmolarity are negligible. Porosity was estimated at 59 ± 3 %. Taking shrinkage into account, the pillars turned out to be thinner than in the original design, and the channels wider.

PTMC-tMA scaffolds, have a structure much stiffer than brain tissues (which shear modulus typically ranges from 0.4 to 1.4 kPa [38]), and were directly inserted in the lesion site without issues. PEGDA-GelMA and GelMA scaffolds were more fragile, and thus more challenging to insert than those made of PTMC-tMA. Yet, the procedure was successful, and allowed to maintain their overall structure, as confirmed by MRI images (Fig. 4). PDSII threads were rigid and very easy to insert.

The intrinsic characteristics of each biomaterial, which enable more or less fine structures to be printed, influenced porosity, which was best for PTMC-tMA (59 %), then PEGDA-GelMA (40 %) and finally GelMA (26 %). T2-weigted images estimated that scaffold volumes were slightly reduced after implantation for PTMC-MA and PEGDA-GelMA (14 % and 19 % respectively) whereas they were drastically reduced for GelMA (48 %) (Table 2). This may affect the real porosity. Scaffold volumes on MR T2-weighted images did not change during the three weeks after implantation (Table 2). T2 MRI PTMC-tMA intensity did not change whereas that of PEGDA-GelMA and of GelMA slightly diminished. This suggests that degradation might begin for the last two biomaterials. Another parameter could also contribute as the liquid into the pores may be replaced by invading cells.

### 3.2 Normal recovery after scaffold implantation

**Fig. 3:**
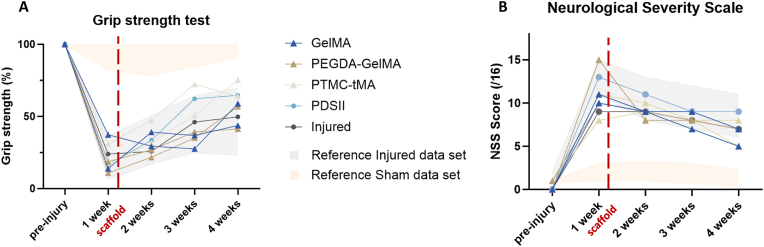
**Behavioral tests**. (A) Grip strength test of the dominant front paw, contralateral to the injected hemisphere, compared to the pre-injury value, expressed in percent. (B) The NSS score shows sensorimotor deficits after malonate injury (score rated out of 16). The graphs show the score curves obtained for each animal in each group at the different time points of the protocol. GelMA: n = 2; PEGDA-GelMA: n = 2; PTMC-tMA: n = 2; PDSII: n = 1; Injured: n = 1. The grey and beige bands represent data sets from Injured (n = 8) and Sham (n = 8) reference groups at 1 month, respectively.

The principal objective of behavioral testing was to assess the animals' recovery post-injury and post-implantation as well as their well-being. As in our previous feasibility study [8], the behavioral tests, conducted blindly, reflect a typical recovery pattern for animals after implantation (Fig. 3). Specifically, implantation of GelMA, PEGDA-GelMA and PTMC-MA did not induce a new drop in sensory-motor functions and allowed a normal recovery (Fig. 3).

**Fig. 4:**
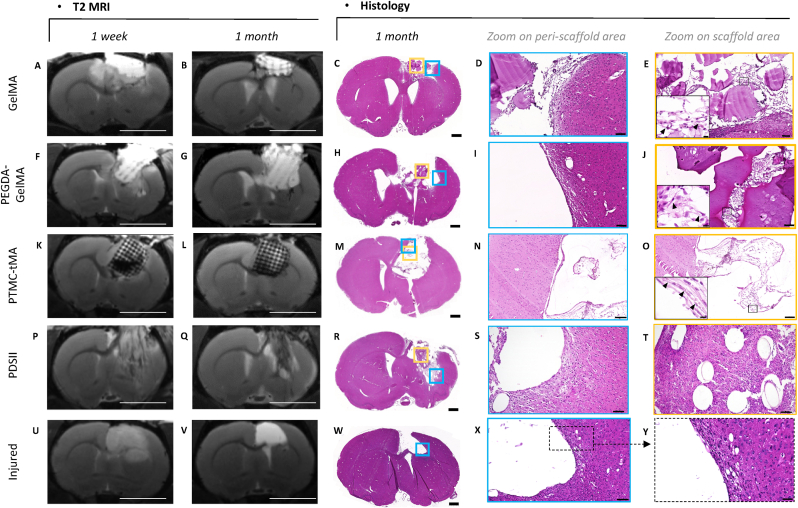
**T2 MRI and histological sections of control and implanted lesioned rat brains.** The left panel showed T2 MRIs at one-week and one-month post-implantation, respectively. The right panel showed hematoxilin-eosin-stained sections of rat brains at 1 month. (A–B) T2 MRIs after the implantation of GelMA, bregma 0.48 mm. (C) GelMA rat brain HE sections showing the fragmented scaffold at the lesion site, bregma 0.48 mm. (D–E) Focus on the peri-scaffold and GelMA scaffold area, respectively. Perilesional brain tissue showed a chronic inflammatory reaction and granulation tissue composed of macrophages, fibroblasts and capillaries. (F–G) T2 MRIs after the implantation of PEGDA-GelMA, bregma 0.24 mm. (H) PEGDA-GelMA rat brain HE sections showing part of the scaffold at the lesion site, bregma 0.24 mm. (I–J) Focus on the peri-scaffold and PEGDA-GelMA scaffold area, respectively. Perilesional brain tissue showed a weak chronic inflammatory response and granulation tissue comprising macrophages, fibroblasts and capillaries. (K–L) T2 MRIs after the implantation of PTMC-tMA, bregma 0.84 mm. (M) PTMC-tMA rat brain HE sections showing tissues that have migrated into the scaffold at the lesion site, bregma 1.08 mm. (*N*–O) Focus on the peri-scaffold and PTMC-tMA scaffold area, respectively. Perilesional brain tissue showed a weak focal chronic inflammatory reaction. (E/J/O) 40x magnification on capillaries (black arrow). (P–Q) T2 MRIs after the implantation of PDSII sutures, bregma 0.24 mm. (R) PDSII rat brain HE sections, bregma −0.00 mm. (S–T) Focus on the peri and suture threads area respectively. (U–V) T2 MRIs after the lesion, bregma 0.24 mm. The hyperintense lesion stabilized after 1 month. The ventricle is dilated due to striatum atrophy. (W) 1-month control rat HE brains section, bregma 0.96 mm. (X) The peri-injured brain tissue shows a thin, stabilized reactive meshwork in contact with the lesion, with few vessels. Normal brain tissue can be seen at a distance all around the injured area. (Y) Boxed area in X, magnification showing no colonization of the lesioned area by cells Scale bar: 5 mm (MRI); 1000 μm (left 20X histology); 100 μm (for 20X histology zoom middle and right) except for (X), 50 μm. HE: hematoxylin-eosin.

The grip test measures the grip strength of the forepaw contralateral to the injured hemisphere, expressed as a percentage of the pre-injury value. Uninjured rats receiving PBS instead of malonate during surgery (Sham) had a relatively constant strength (Fig. 3A-beige band corresponds to the reference sham data set from previous experiments). After malonate injection, injured rats (grey band, reference injured data set from previous experiments) without scaffold showed a significant loss of strength in their dominant paw, giving a 19.8 % grip performance. After one month, the spontaneous recovery after lesion was around 43.6 % of grip strength.

The two control injured rats, one without scaffold and the other with PDSII threads, displayed a normal recovery at the grip strength test and NSS score. Their behavioral curves were consistent with those from the reference injured data set (Fig. 3). The curves of the implanted rats also followed within this recovery interval. It should be noted that the scaffolds implantation can cause localized damage responsible for slight latency or weakness in post-injury recovery.

The same results were obtained for the NSS, which showed stable sensorimotor performance for the reference Sham group. Sensorimotor deficits of 12.7 score were observed following malonate injection in the reference injured group (Fig. 3B-grey band). The curves of the implanted rats followed the post-injury recovery interval.

The same results were found for the number of elevations. Rats displayed 8.8 ± 2.9 elevations in 5 min in the open field before the lesion. Three weeks after implantation, implanted rats showed a decreased performance of 56.1 ± 28.3 % that did not differ from the control lesioned rat at 48 %.

### 3.3 Scaffolds did not cause major inflammation in injured rat brain

The control rat had a clean lesion edge and a thin reactive band in contact with the lesion (Fig. 4, X,Y). As an additional control of the standard inflammatory reaction to expect after a lesion and the introduction of a foreign material known to be well tolerated, we included in our comparative pilot study a rat implanted with PDSII. The 4-6 mm-long sutures appear in MRI as a hypointense claw-like shape going from the motor cortex to the striatum (Fig. 4 P-Q). Histological analysis of brain sections stained with hematoxylin-eosin revealed that the threads implanted were still present 1-month post-lesion (Fig. 4, R-T), both within the lesion and perilesional area, which included pre-existing brain tissue. In the post-necrotic zone, the reconstructing tissue was quite sparse. It was neovascularized, with capillary, venous and arterial vessels. There was very little reaction around the wire residues, some of which were surrounded by few inflammatory cells, notably macrophages, responsible for biomaterial disposal. This foreign-body reaction remained minimal and localized, confirming that, as we expected, the PDSII was well tolerated. However, this type of physiological reaction is common and considered acceptable in human clinical practice, and will serve as a reference for analyzing the cerebral biocompatibility of other biomaterials.

One major result of this study lies in the persistence of the biomaterials, in the brain 1-month post-implantation. In this context, T2 MRI follow-up is highly informative. Indeed, all the biomaterials were visible on T2 MRI from implantation up to 1-month post-implantation and displayed their initial shape (Fig. 4). GelMA and PEGDA-GelMA were T2-hyperintense, while PTMC-MA was T2-hypointense. T2 MRI therefore enables non-invasive monitoring of scaffold degradation over time. Histological analysis at 1 month revealed results congruent with pre-sacrifice imaging at 1 month (Fig. 4). A parallel can be drawn between the pre-sacrifice MRI images and the histological sections at 1 month. MRI sections are more representative of the correct scaffold position within the brain prior to sacrifice than post-mortem histological section. Histology may result in material loss, deformation or biomaterial offset during cutting. We found that GelMA still persisted in the brain at one month, with a relatively well-preserved architecture. The hyperintense scaffold covered the cortical lesion zone, and exhibited clear architectural patterns (Fig. 4 A-B). As a protein, gelatin takes on a violet color, unlike the other materials we tested. Histological analysis revealed a more fragmented and collapsed scaffold than seen in MRI, but these differences likely result from the histological technique. This soft, fragile gelatin scaffold is sensitive to the cutting and heat involved with the histology protocol. Cell migration was limited to the periphery of the scaffold, with a network of small vascularized cells loaded with macrophages (Fig. 4. E). Due to the rigidifying role played by PEGDA, the PEGDA-GelMA implant was stiffer and resisted better to histological techniques, showing more preserved 3D patterns. In the implant, the loose fibrous tissue was neovascularized, with few macrophages. The biomaterial was dotted with small degradation vacuoles (Fig. 4. J). Finally, PTMC-tMA, which is even more rigid and therefore more resistant to friction and degradation, presented a general architecture very close to the original CAO design, with preserved geometric patterns. The empty space within the implant was invaded by a fine vascularized network (Fig. 4. O). Nevertheless, the material rigidity proved to be a problem for the histological technique, particularly during cutting. The mechanical difference between tissue and PTMC-MA scaffold caused severe damage to the sections as the blade passed over them. Only a few brain sections were obtained with the scaffold. On Hematoxylin-eosin (HE) slices, all the printed biomaterials allowed the colonization of the scaffolds, presenting endogenous cell invasion and neovascularization at 1 month (Fig. 4).

⁃ Tissue reaction: Glial and fibrotic response

**Fig. 5:**
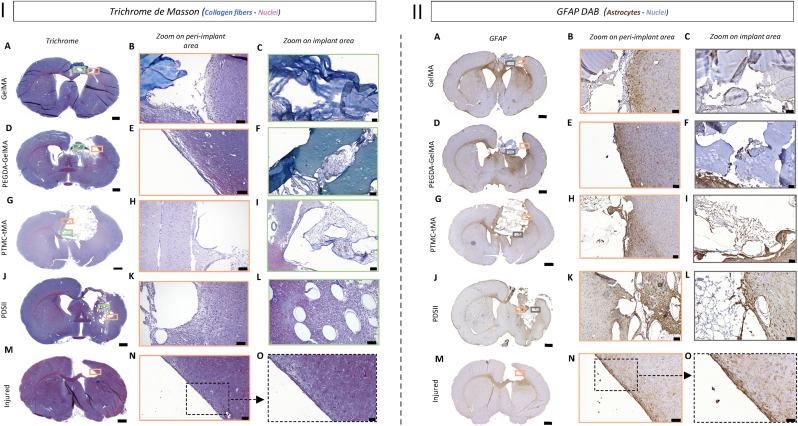
I - **Histology of trichrome stained brain sections and characterization of perilesional and implanted areas**. (A, D, G, J, M): topography of lesions at low magnification on coronal sections stained with Masson's trichrome (left). The collagen deposit is identified by the blue coloration surrounding the scaffolds. Higher magnification shows perilesional brain tissue (orange boxed area) and peri-scaffold reactions (green boxed area). For GelMA (B–C), the peripheral staining is weak and physiologic with few loose fibers in the tissue, and collagen rings around the vessels can be seen. For PEGDA-GelMA (E–F), the staining is weak and physiologic with few loose fibers in the tissue, and collagen rings around the vessels can be seen. For PTMC-tMA (H–I), there was a presence of a fine collagenous mesh at the peripheral tissue-scaffold interface. For PDSII (K–L), normal-appearing peripheral tissue, with only a collagen ring at the suture section edge. For the injured rat (*N*–O), slight reorganization around the lesion, at the forming glial scar level, with few collagen fibers at the lesion edge and collagen around the vessels. II - **Histology of Glial fibrillary acidic protein (GFAP) stained brain sections and characterization of the glial barrier after lesion and implantation.** (A, D, G, J, M): topography of lesions at low magnification on coronal sections stained with Glial fibrillary protein (GFAP) (left). The GFAP deposit is identified by the brown coloration of astrocytes(/glial cells) surrounding the scaffolds. Higher magnification shows perilesional brain tissue (orange boxed area) and peri-scaffold reactions (grey boxed area). For GelMA (B), heterogeneous glial scarring can be seen at the scaffold/host tissue interface. (C) No astrocytic population appears to be present within the implant. For PEGDA-GelMA (E), a discrete glial barrier is visible at the implant/host tissue interface. (F) No astrocytic population appears to be present within the implant. For PTMC-tMA (H), a glial barrier molded to the edges of the scaffold can be seen at the scaffold/host tissue interface. (I) Some astrocytes appear to be present inside the scaffold. For PDSII (K–L), a strong astrocytic reaction visible at the lesion edge with a thin barrier around the sutures, reconstructing tissue poor in astrocytes. For the injured rat (*N*–O), thin glial barrier at the lesion/host tissue interface, astrocytes aligned parallel to the lesion. Scale bar: 1000 μm (left histology (slide scan x20), whole slice); 100 μm (for histology zoom middle and right) except for (O), 50 μm.

In order to assess the biocompatibility of the biomaterials, the lesion edges and the tissue reaction to biomaterial contact were observed. HE, GFAP-DAB and Masson's trichrome stainings showed that none of the biomaterials triggered excessive foreign body reaction or fibrotic encapsulation of collagen (Fig. 4, Fig. 5). The tissue reaction around the GelMA biomaterial did not show signs of inflammation and appeared to be pro-regenerative. GelMA, as a protein, uptakes blue color, but no collagen fibers were visible in the scaffold, which had little tissue colonization (Fig. 5. **I**–C). Indeed, the reconstructing tissue environment was fibro-vascular and very well reticulated, with colonized mature vascular pedicles with collagen fibers, and many small capillaries, arterioles and veins throughout, hemosiderin-pigmented cells (hemosiderophages), few inflammatory cuffs and arterioles with veins throughout (Fig. 4, Fig. 5). Small capillaries were visible, indicating a highly vascularized rather than fibrous framework. In areas where the biomaterial is more present, the reconstructing tissue and its vascular network are less important, since space is still occupied by gelatin. Peripheral patterns are colonized but not the core of the implant.

PEGDA-GelMA showed more consolidated 3D patterns. The 3D space was appropriately colonized by a very fine, loose fibrous matrix in which a few vessels and macrophages were detected. The inflammatory reaction appeared slightly more developed than with GelMA alone, but remained focal. A fine, loose collagenous tissue colonized and filled the empty spaces in the scaffold (Fig. 5I. E-F). HE labeling revealed a lesion core with a higher ratio of reconstructing tissue with PEGDA-GelMA compared to GelMA. Despite its relatively well-preserved structure, PEGDA-GelMA showed signs of degradation, with vacuoles scattered throughout the scaffold material (Fig. 4). Analysis of the synthetic PTMC-tMA revealed highly controlled inflammation, with a very fine fibrotic mesh developing around the biomaterial (Fig. 5. **I**–I). Nevertheless, the patterns appeared to be fairly well colonized, with a weave similar to PEGDA-GelMA. Incipient vascularization was observed between the patterns, with a few scattered macrophages.

⁃ Effects of implanted scaffold on glial barrier formation and microglia activation

Glial cells are essential to the proper functioning of the brain. In this study, we assessed the characteristics of the glial barrier by evaluating astrocytes and the density of microglial cells recruited at the lesion edges and around the biomaterials.

**Fig. 6:**
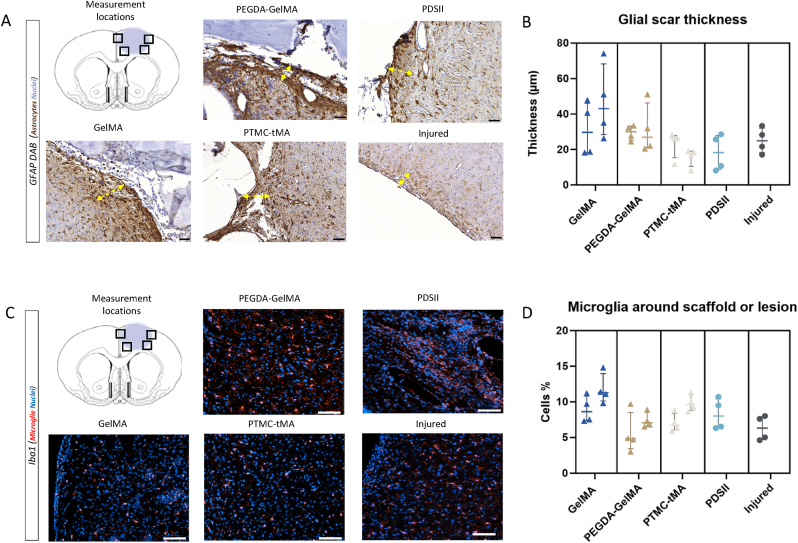
**Characterization of lesion edges: glial barrier and microglia.** (A) Representative images of glial barrier observed on the GFAP-stained sections. (B) Quantification of brains glial barrier thickness. GelMA: n = 2; PEGDA-GelMA: n = 2; PTMC-tMA: n = 2; PDSII: n = 1; Injured: n = 1. Measurements made in 4 independent fields of one representative slice for each rat separated on the x axis. (C) Representative images of the microglial dispersion observed at the lesion edge on the different immunofluorescence sections labelled with Iba1. (D) Estimated number of Iba1+ cells (%) around scaffold or lesion. Measurements made in 4 independent fields for each rat on two different level slices. The points on the graphs represent the data measured per field for each rat separated on the x axis, with their respective medians and interquartile. Scale bar (slide scan x20): (A) 50 μm; (C) 100 μm. GFAP: glial fibrillary acidic protein.

Glial barrier thickness was measured using rat brain sections representative of the respective lesions in each animal. Four independent fields per rat, in four equivalent anatomical areas on a representative section (Fig. 6-A) were selected to determine mean barrier thickness using a macro integrated into ImageJ software. The results are presented on the graph (Fig. 6-B), with medians and interquartile for each biomaterial. There was some variability between the different biomaterials, but none of these differences were significant, and overall, no biomaterials produced excessive glial scarring compared to controls. A slight variability in thickness is observed for GelMA, reflecting the heterogeneity of the glial barrier around this biomaterial. The median thickness measured for PDSII of 18.20 μm is close to the others biomaterials tested: PTMC-tMA: 18.80 μm; PEGDA-GelMA: 29.75 μm and finally GelMA: 37.90 μm.

Scaffold implantation did not exacerbate the inflammatory response in the brain, as evidenced by Iba1 labeling of microglia and macrophages (Fig. 6-C/D). Four immunofluorescence fields were taken to quantify and average across slices to obtain a percentage of Iba1-positive cells at the lesion edges. There were no significant differences between the different groups (Fig. 6-D). Thus, biomaterials did not generate enhanced microglial responses compared to the PDSII control or the non-implanted injured rat. Biomaterials, such as PEGDA-GelMA or PTMC-tMA, even appeared to stabilize the microglial response, with a slightly smaller population around the lesion (median: Injured: 6.3 %, PEGDA-GelMA: 6.6 % and PTMC-tMA: 8.7 %).

⁃ Scaffold colonization

**Fig. 7:**
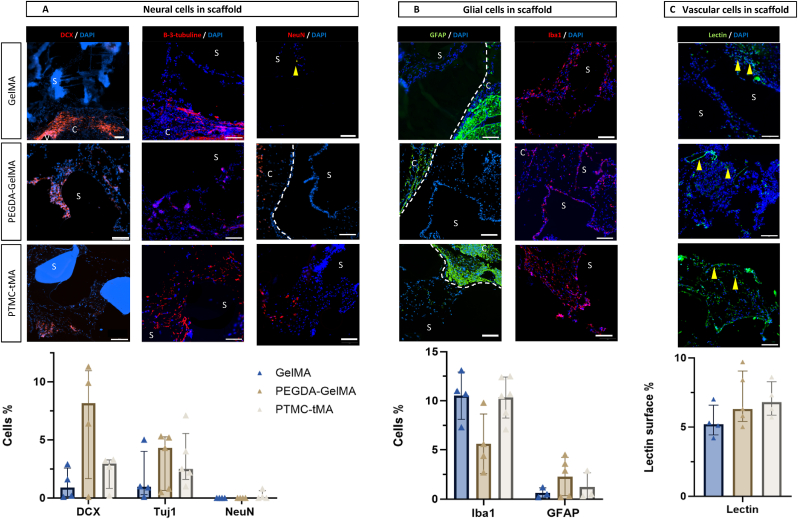
**Characterization of colonizing tissue in the scaffold: Neural, glial and vascular cells.** Representative scaffold images for each marker. Measurements taken in 3–6 independent fields per rat on two to three slices. One graph per estimated cell type (A, B, C). The points on the graphs represent the data measured per field for each rat, with their respective medians and interquartile. Scale bar: 100 μm, 20X magnification. S: Scaffold; C: Cortex; V: Ventricle; DCX: Doublecortin; GFAP: glial fibrillary acidic protein.

The aim of biodegradable scaffolds is to provide support and anchorage for cell development, in order to recolonize the lesion where the extracellular matrix has been destroyed. It is therefore important to look at scaffold colonization at 1 month, in order to anticipate the quality of the regenerative capacity of these scaffolds in the long term. Visually, as mentioned above, the GelMA scaffold showed colonization mainly in the periphery, with only few cells in the GelMA core (Fig. 7). In contrast, the other two scaffolds showed more diffuse and deeper colonization (Table 5). Cell spreading in the scaffolds was estimated on 3 section levels. A grid was superimposed on the scaffolds, and a percentage of the number of colonized grid squares was extracted. Colonization, and thus cellular density, was similar for all printed biomaterials. PTMC-tMA and PEGDA-GelMA in a lesser extent allowed the best colonization spreading compared to GelMA.

**Table 5:** Scaffold colonization.

Biomaterials	Porosity (%)	Spreading (%)	Cell density in scaffold pores (cells/mm^2^, DAPI+) ± Deviation from average
**GelMA**	26	30	4748 ± 1658
**PEGDA-GelMA**	40 ± 6	65	3434 ± 750
**PTMC-tMA**	59 ± 3	85	3104 ± 851

Quantification of the number of cells in the pores carried out on 6 to 9 independent fields. For colonization spreading, a grid was superimposed on the scaffolds, and an approximate ratio of the number of grid squares with colonizing tissue to the number of empty grid squares was extracted. For the number of cells relative to the surface area in the pores, quantifications of the number of DAPI + nuclei were performed on the fields corresponding to the colonized pores using ImageJ software. The mean density of cells in the pores is given for each biomaterial.

### 3.4 Tissue characterization

Once we confirmed the colonization of the scaffolds by a neo-tissue, it was interesting to characterize it for each implanted group. The tissue was labelled with various specific immunofluorescence markers to determine the different cell types present in the scaffolds, neuronal, glial and vascular types.

The neuronal cell type has been identified by the markers DCX, β-3-Tubulin and NeuN, which marked neuronal progenitors, immature neurons and mature neurons respectively. The glial types will be represented by the aforementioned markers Iba1 and GFAP, which identified microglia and astrocytes respectively. Finally, neovascularization was highlighted by the lectin marker. For each marker, 3 to 6 fields on 2 to 3 different slice levels, depending on the material available, were acquired and quantified for each printed biomaterial.

The GelMA scaffold, colonized only at its periphery and weakened by the technique, showed few pores compatible with good characterization. The subventricular neurogenic niches (SVZ) were highly activated and very rich in DCX+ and β-3-Tubulin + cells. Nevertheless, only a few cells migrating from the neurogenic niche were detected into GelMA patterns with 0.9 % of DCX+ and 0.9 % of β-3-Tubulin + cells (Fig. 7A). In the case of PEGDA-GelMA, 8.1 % were DCX+ and 4.3 % of beta-3-tubulin cells were identified in the implant. Finally, PTMC-tMA showed an intermediate behavior, with medians of 2.9 % and 2.5 % respectively (Fig. 7A). No mature neurons characterized by NeuN were found in the neo-tissue for all the scaffolds. Although a definite migration of neural progenitors into the scaffold has been identified, it should be noted that differentiation into mature neurons was not confirmed in these structures at 1 month.

For the glial population inside the scaffold, there was no significant difference in the percentage of Iba1+ cells in the structure (Fig. 7B). The GelMA and PTMC-tMA scaffolds had similar percentages of Iba1+ cells, while the PEGDA-GelMA scaffold appeared to have half of identified microglia in contact with the biomaterial. Surprisingly, the scaffolds were devoid of astrocytes. Only a few peripheral pores seemed to reveal a few GFAP + cells in some structures, but nothing notable (Fig. 7B).

Finally, although achieving clear labeling in all gaps was challenging, vascular cells were abundant throughout the scaffold's pores. Following all directions and presenting varying levels of maturity, neovessels represented on average 5–7 % of the colonizing tissue of scaffolds whatever the biomaterial tested (Fig. 7C). This result was comparable to the lectin labeling in the healthy cortex (6.6 ± 1.4 %). Arterial Spin Labeling MRI was not sensitive enough to detect perfusion in reconstructing tissue with emerging neovascularization in implanted rats (Suppl Fig. 2). However, no abnormal perfusion was seen in contact with the scaffolds.

## 4 Discussion

In this *in vivo* study, we investigated the biocompatibility of GelMA, PEGDA-GelMA, PTMC-tMA and P(PF-MCL-PF) for brain injury. The last two were tested for the first time in the brain. These photoprintable biomaterials, described as bioactive and slowly degradable [11,19,[39], [40], [41]] were printed as 3D regenerative scaffolds for brain repair. To date, accurate data on their behavior *in vivo*, and specifically in the brain, are scarce; therefore, a first comprehensive biocompatibility evaluation appeared necessary. Our feasibility study evaluated these biomaterials, covering the entire process from the printing ink formulation to the histological analysis after their implantation into the injured rat brain. Printing was performed based on a 3D model previously described in detail [8], designed to enable 3D tissue reconstruction in three privileged directions. Such a complex 3D structure suited for cortical and brain architecture requires a printing resolution that is not achievable with all biomaterials. The scaffold design was adapted according to the printing resolution of the different biomaterials and the characteristics of the respective solvents. GelMA is viscous under 37 °C, and its viscosity is highly dependent on the printing temperature, making GelMA printing by DLP quite complex. PEGDA, which is easier to print, made it possible to adjust the PEGDA-GelMA printing once the two biomaterials had been properly homogenized. PTMC-tMA proved much more easy to print, and propylene carbonate solvent used in the formulation, is ideal for DLP printing due to its high boiling temperature [42]. Unfortunately, P(PF-MCL-PF) is poorly soluble, and ethyl acetate solvent gradually evaporates during DLP printing. This compromised the printing process, making it impossible to fabricate 3D porous architectures. This limiting step affected the comparison with other biomaterials. Despite the technical hurdle, non-patterned photoprinted P(PF-MCL-PF) fragments were implanted into the injured brain to get a first idea of the physiological tissue reaction.

Intracerebral implantation is a crucial step. In injured animals, cerebral edema increases intracerebral pressure, and invasive procedures may exacerbate tissue damage. The challenge is twofold: the scaffold must not collapse on itself during implantation and must retain its original structure, without causing further damage to the surrounding tissue. In our study, the ease with which the biomaterials could be implanted was proportional to their respective rigidity. The low stiffness of GelMA, approximately 22 ± 5 kPa (CellInk), was found to be the threshold for implantability. Post-implantation MRI allowed to check that scaffolds had been properly implanted, they appeared hyper-intense (PEGDA-GelMA and GelMA) or hypo-intense (PTMC-tMA and P(PF-MCL-PF)) on T2 MRI whether or not they contain water. Fortunately, all scaffolds, even GelMA, the more fragile one, retained their porous geometries. T2-weighted MRI allowed *in vivo* longitudinal monitoring of porosity and cerebral acceptability of scaffolds. The resolution was sufficient to see the structure, quantify scaffold volume and intensity (Table 2). But it was difficult to correlate variations in scaffold intensity and degradation since cell invasion into the pores could also be an explanation of the slight decrease in intensity for GelMA and PEGDA-GelMA implants. However, it did provide essential information that GelMA scaffolds may have lost much of its porosity.

Longitudinal behavioral analysis with validated tests [8,35] was used to monitor clinical signs and post-operative recovery in implanted animals, and was compared to known standard recovery profiles. No worsening of deficits or side-effects associated with the scaffolds were observed, and no clinical side-effects related to the biomaterial were observed, suggesting that implantation of these scaffolds was well-tolerated in injured animals.

For histological analyses, to preserve scaffolds implanted into the brain, we chose to embed brain tissue in paraffin. Consequently, samples were subjected to chemical, thermal and mechanical perturbations that must have had some impact on the biological material. GelMA, fragile and heat-sensitive, showed highly fragmented and disordered patterns at histology, whereas pre-sacrifice MRI shows structural preservation. Coupling GelMA with a stiffener could make it less susceptible to deformation as we did with PEGDA. PTMC-tMA was most of the time too rigid to be well cut. It would be interesting to couple PTMC-tMA with other materials, like PEG, to decrease its stiffness and facilitate its slicing [16]. Overall, the PEGDA-GelMA composite provides a well-balanced combination of gelling and stiffening properties, which deliver the most stable and preserved structure after the histological processing.

Biocompatibility and tissue regeneration are assessed by means of specific cyto-architectural patterns on the biomaterial. During the repair process, two distinct phenomena occur: fibrosis and tissue regeneration. An inflammatory response is to be expected, whatever the degradable material introduced. Scaffolds, which may degrade slowly, will progressively induce an inflammatory reaction characterized by the prevalence of macrophages, known as granulomatous inflammation [43]. To establish a benchmark of minimal acceptable inflammation, we used a control rat for the inflammatory reaction in which we introduced intracerebral PDSII degradable sutures, commonly used in clinical practice today [44]. The rate of inflammation around and within the scaffolds (5–10 % of Iba1+ cells) was found similar than those reported in the literature [45].

When brain tissue is damaged, astrocytes are recruited to form a glial barrier, preventing the lesion from spreading to healthy peripheral tissue [46]. When the foreign agent is considered too harmful for the brain, it may be encapsulated in a collagenous fibrous shell [45,47]. Our study revealed a similar tolerance of the biomaterials tested compared to PDSII sutures. The collagenous fibrosis, glial scarring and microglial population surrounding the materials were low and comparable to those found for PDSII, indicating that implanted biomaterials were well accepted by the brain tissue. Moreover, the average thickness of glial scarring found around the materials was comparable to the injured control rat and was even less to other studies [35,[48], [49], [50]]. The glial barrier must be thin and permissive, and is a plastic barrier, which both protects healthy tissue by acting as an insulator from the injured site [51], and sustain the regeneration by acting as a selective filter permeable to endogenous cells [52]. In this study, the relatively thin, permissive scar of the PEGDA-GelMA and PTMC-tMA scaffolds enabled remarkable scaffold colonization. The slightly greater barrier thickness of the GelMA scaffold did not make it impermeable, since migration beyond the glial barrier was observed.

Together with the material biocompatibility, the tissue colonization of the implanted scaffold is of paramount importance. The biomaterial is not used to fill a cavity, but to regenerate it. To achieve this, the porous structure must host enough endogenous cells to initiate the formation of an organized and diversified neo-tissue. In the present study, the colonization rate of all implant patterns 3 weeks after implantation ranged from 30 % to 85 %, depending on the biomaterial with a mean cell density of 3.7 ± 1 .10^3^ cells/mm^2^. The biomaterial colonization can depend both on the attraction of the material itself and on its porosity [53]. We found colonization by endogenous cells occurred differently depending in the biomaterials. GelMA was fragile, with the lowest channel width (225 ± 42 μm), and lowest porosity (26 % but much less after implantation) and worst colonization, colonized only at its periphery. Despite its weakness and limited porosity, the pre-sacrifice MRI scan showed preservation of the structure and of some pores. Thus, the rate of GelMA degradation was not a crucial factor at this stage, since porosity was somehow preserved. On the other hand, it's true that colonization at the periphery but not at the core suggests a migration defect, counterintuitive to the literature [54,55]. The concentration of GelMA could be one explanation [56]. Indeed, the higher the GelMA concentration in the hydrogel, the lower the adhesion, proliferation and extension of neurites. However, 10 % seems to be the best compromise between adhesion, spreading and neurite length [56]. CellInk GelMA hydrogel, at around 10 %, is consistent with the optimal neurite spread and growth. Mechanical properties are probably not responsible for the inhibition of cell migration, as different rigidities allow cell colonization [3,57]. Interestingly, cross-linking may be an explanatory factor since Macaya et al. showed inhibition of cell infiltration in a crosslinked hydrogel. Interestingly, this was reversed by a growth factor (FGF-2) [58]. Although GelMA retains some RGD sites, they may be insufficient to trigger cell attraction, particularly in the absence of active chemotactic factors (e.g. CXCL12/SDF-1α, FGF2, MCP-1) known to be crucial for SVZ migration to its target [59].

PEGDA-GelMA appeared highly colonized in the majority of the patterns still visible on histology. The pores appeared to be filled with molded tissue at the edges of the degrading biomaterial. Of course, further data on its long-term cerebral integration and regenerative capacity are still required. PTMC-tMA did not show signs of degradation and showed complete colonization of the pores both at the periphery and in the center.

The nature of colonizing tissue and a perfect symbiosis between neuronal, glial and vascular cells may provide information for a future gain in function. The neuronal population within the scaffolds showed a maturation profile. We found more neuronal progenitors, slightly fewer immature neurons and almost no or no mature neurons. Progenitor maturation did not appear to be complete. In general, complete differentiation of neural progenitors (DCX+) into mature neurons (NeuN+) takes approximately 4–6 weeks after migration into the scaffold [60,61]. An inflammatory microenvironment can partially slow maturation [62]. At 1 month, as in our study, the context is not yet totally favorable. This study aimed at characterizing the acute inflammatory reaction. A longer study could enable us to check whether neuronal maturation continues beyond 1 month. Firstly, it was imperative to study the base material to ascertain whether the biomaterial itself was either limiting (PEGDA or GelMA) or limited (composition too far from the complexity of the matrix). Secondly, the process of neuronal differentiation may be enhanced by the application of a coating, such as laminin or hyaluronic acid, as well as the addition, if necessary, of maturation factors [58].

Surprisingly, few astrocytes colonized all scaffolds and this may have an impact on neuronal maturation. It will be pertinent to decipher whether it is due to a short 1-month period of evaluation or to a rejection by the biomaterials. A decrease of astrocytic colonization has already been described for spinal cord injury scaffold made of gelatin, which was accentuated when gelatin was methacrylated (GelMA) [39]. However, it has to be noted that such effect was not observed for poly(acrylate) copolymer [2,63]. Furthermore, astrocytic attraction can easily be improved by functionalizing our scaffolds with laminin, fibronectin or YIGSR peptides, or also by loading chemoattractants such as CXCL12, VEGF or FGF2 into the gel [58]. In addition, gradient-based delivery can be envisaged to simulate directional wounding signals.

More and more neuro-regeneration studies are coupling cell therapy and biomaterial engineering. Adding cells is probably the most effective strategy for attracting other cells, since cells secrete extra-cellular matrix components and produce growth factors. But this complicates translation to humans, which is why a cell-free solution is sought here.

Last but not least, the vascularization of scaffolds is the first requirement for cellular colonization. At 1-month, perilesional perfusion assessed with MRI was normal around the scaffolds. However, this technique was not suited to correctly assessed a perfusion in a tissue in reconstruction. Intra-scaffold vascularization was quantified as normal (5–7 %), with no difference between the different biomaterials. The biomaterials tested in the present study favored a vascularization of the tissue in reconstruction similar to the one induced by a growth factor in the same brain lesion model [19].

In conclusion, considering the advantages and disadvantages of each biomaterial, PEGDA-GelMA showed the most favorable characteristics. In the long term, it could play as promising a role in the brain as it does in the spinal cord, where, loaded with neural progenitor cells, it has demonstrated a functional efficacy [6]. Indeed, PEGDA-GelMA might serve as a base biomaterial that can be used as such for cell support or enhanced with extracellular matrix components and/or mixed with cells for brain and spinal cord regeneration. GelMA, which was difficult to insert into the brain without breaking, retained its architecture *in vivo*, but did not stand up well to the histological technique. Cell migration was essentially peripheral, with a scaffold core devoid of endogenous tissue. PTMC-tMA was easier to insert but posed technical problems for histology and can potentially increase intracerebral shear forces. The tissue reaction around the light-cured P(PF-MCL-PF) pieces encourages us to pursue evaluation of 3D porous scaffold for brain regeneration and if no regeneration would occur, it could be used as a safe aesthetically filling material, largely well tolerated. Above all, PEGDA-GelMA composite was the most ideal candidate for intracerebral implantation. In addition to its biophysical and bioprinting qualities, it facilitated the creation of a permissive glial barrier, induced neovascularization and attracted neuronal progenitors. In the future, a targeted, longer and more comprehensive study will provide the additional elements needed to better understand the attraction mechanisms underlying PEGDA-GelMA scaffold.

## Author contributions

MC: experimental work, analysis and interpretation of data, writing original manuscript. NC: experimental work, data analysis, editing. JC: design and printing of scaffolds. FD: MRI acquisition, data analysis and interpretation, editing. AB: analysis and interpretation of MRI data, editing. JF: Gal-C9 inventor and manufacturer, editing. MC: Gal-C9 preparation. MB: P(PF-MCL-PF) inventor and manufacturer, editing. SB: PTMC-tMA inventor and manufacturer, editing. LR: histological expertise. MP: histopathology experiments. IRL: histopathology analysis and interpretation, editing. CC: immunofluorescence analysis and interpretation, funding acquisition, manuscript revision, supervision. IL: study design, data analysis and interpretation, funding acquisition, manuscript revision, supervision. All authors contributed to the article and approved the submitted version.

## Funding

This work was supported by 10.13039/501100022278Foundation “Gueules Cassées” [grant numbers 70-2019, 23–2019, 24–2020, 24–2021, 55–2021, and 22–2022], Agence Innovation Défense [JC], and Agence Nationale de la Recherche (ANR), France [Grant number ANR-19-ASTR-0027]. This work, bearing the reference EUR CARe N°ANR-18-EURE-0003, has benefited from State aid managed by the Agence Nationale de la Recherche under the Programme Investissements d'Avenir. We acknowledge the support of ECELLFrance "The national research infrastructure for regenerative medicine - MSC-based therapies" (France 2030/ANR-24-INSB-003) for the LabHPEC/Melissa Parny.

## Declaration of competing interest

Nothing to disclose.
