# Supplementary material for: Printable biomaterials for 3D brain regenerative scaffolds: An in vivo biocompatibility assessment
Source: Regen Ther. 2025 Aug 19;30:641–55. doi: 10.1016/j.reth.2025.08.008 (PMC12395985; doi:10.1016/j.reth.2025.08.008)
Supplement: Multimedia component 1 [file mmc1.pptx]

## Slide 1
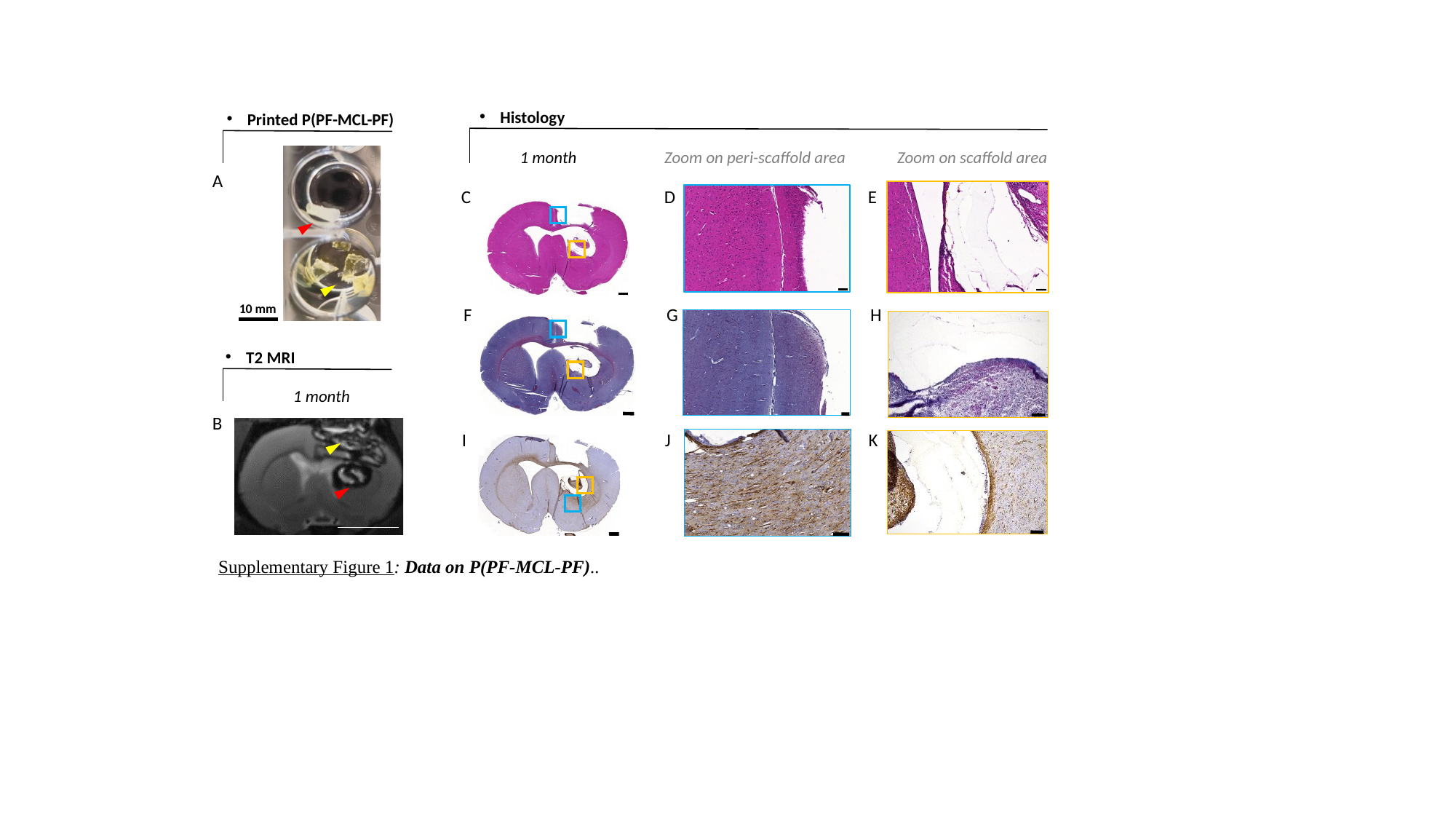

Histology
Printed P(PF-MCL-PF)
1 month
Zoom on peri-scaffold area
Zoom on scaffold area
A
C
D
E
F
G
H
10 mm
T2 MRI
1 month
B
I
J
K
Supplementary Figure 1: Data on P(PF-MCL-PF)..

## Slide 2
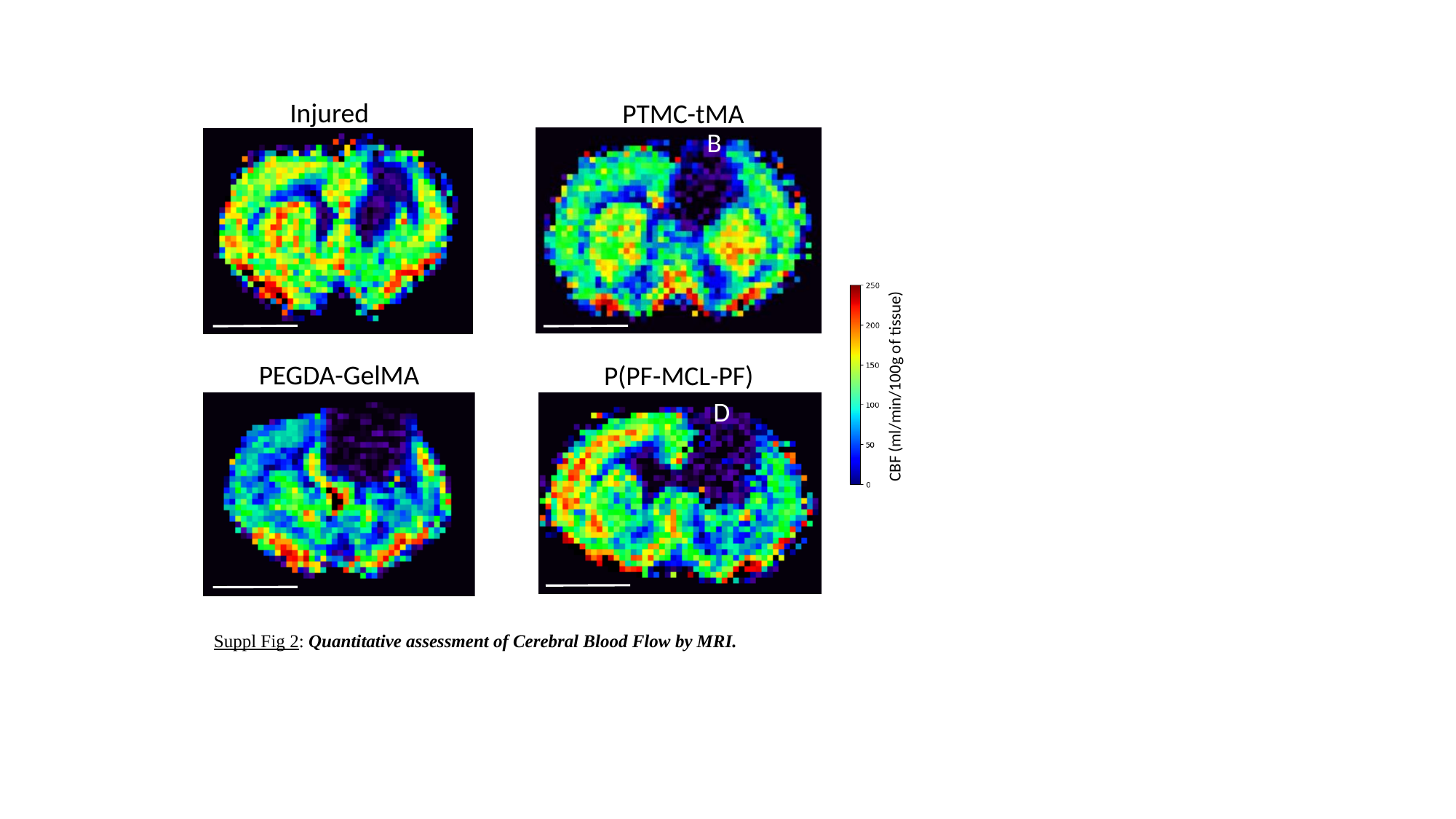

Injured
PEGDA-GelMA
				 PTMC-tMA
				 P(PF-MCL-PF)
A						B
CBF (ml/min/100g of tissue)
C						 D
Suppl Fig 2: Quantitative assessment of Cerebral Blood Flow by MRI.
