# Supplementary material for: Printable biomaterials for 3D brain regenerative scaffolds: An in vivo biocompatibility assessment
Source: Regen Ther. 2025 Aug 19;30:641–55. doi: 10.1016/j.reth.2025.08.008 (PMC12395985; doi:10.1016/j.reth.2025.08.008)
Supplement: Multimedia component 2 [file mmc2.docx]

Supplementary Figure 1: **Data on P(PF-MCL-PF)**. (A) Top view of a well plate containing pieces of photocrosslinked P(PF-MCL-PF). In the top well, a rectangle of material can be seen rolled up into a kind of cylinder, red arrow at A and B. (B) Brain T2 MRI 1 month after a large lesion and 3 weeks after the implantation of P(PF-MCL-PF) showing various biomaterial tips at cortical level above the P(PF-MCL-PF) cigar, section at bregma 0.12mm. The P(PF-MCL-PF) is hypointense. (C-K) P(PF-MCL-PF) histology: brain sections at 1 month labelled with HE (C-E), Masson’s Trichrome (F-H) and GFAP (I-K). Scale bars: 1000μm.

HE: hematoxylin-eosin, GFAP: Glial fibrillary acid protein.

Supplementary Fig 2: **Quantitative assessment of Cerebral Blood Flow by MRI** for the injured control rat (A), PTMC-MA rat (B), PEGDA-GelMA rat (C), and P(PF-MCL-PF) rat (D). Brain perfusion assessment shows the hypoperfused lesion and implantation areas one month after the lesion. Scale bar: 5 mm.
